# Benchmarking TCR–pMHC structure prediction: a unified evaluation and CDR3-based functional insights

**DOI:** 10.1093/bib/bbag289

**Published:** 2026-06-05

**Authors:** Jiadong Lu, Xinyuan Zhu, Xinting Hu, Cheng Zhang, Fuli Feng

**Affiliations:** School of Artificial Intelligence and Data Science, University of Science and Technology of China, Huangshan Road, Shushan District, Hefei 230027, Anhui, China; Zhongguancun Academy, No. 17, Second Ring Road, Daniufang, Haidian District, Beijing 100094, China; School of Information Science and Technology, University of Science and Technology of China, Huangshan Road, Shushan District, Hefei 230027, Anhui, China; School of Artificial Intelligence and Data Science, University of Science and Technology of China, Huangshan Road, Shushan District, Hefei 230027, Anhui, China; Bioscience and Biomedical Engineering Thrust, The Hong Kong University of Science and Technology (Guangzhou), Duxue Road, Nansha District, Guangzhou 511458, Guangdong, China; School of Artificial Intelligence and Data Science, University of Science and Technology of China, Huangshan Road, Shushan District, Hefei 230027, Anhui, China

**Keywords:** TCR–pMHC, AlphaFold, protein structure prediction, benchmark

## Abstract

Interactions between T cell receptors (TCRs) and peptide-major histocompatibility complexes (pMHCs) are central to adaptive immunity. Recent advances in structure prediction tools have enabled atomic-level modeling of TCR–pMHC interactions. However, the lack of systematic evaluation forces practitioners to invest substantial resources in selecting appropriate tools. Here, we present a comprehensive benchmark of TCR–pMHC structure prediction with 70 previously unseen complexes and 13 models spanning MSA-based, PLM-based, and docking-based approaches, revealing the superior modeling accuracy and docking quality of MSA-based methods, especially AlphaFold3. To further enhance the utility of AlphaFold3 predictions, we identify the pLDDT score of the TCR CDR3 region as an informative indicator of both structural correctness and functional relevance. Specifically, it enables up to 4.3% Top-1 success gain through reranking and captures mutation-induced affinity changes in 75.3% of cases. Overall, our analysis would facilitate the practical usage of immune structure prediction models and guide the advancement of these models.

## Introduction

The adaptive immune system, critically orchestrated by T cells, serves as a frontline defense against an array of threats, from rapidly evolving pathogens to cancerous cells [1, 2]. At the core of this defense, T cell receptors (TCRs) recognize antigenic peptides presented by major histocompatibility complex (MHC) molecules. These interactions form TCR–pMHC complexes that govern the specificity and breadth of immune responses. Understanding the structural basis of TCR–pMHC interactions is essential for uncovering the principles of immune recognition [3, 4] and for enabling applications such as vaccine development and personalized immunotherapy [5, 6]. However, experimental determination of TCR–pMHC complex structures remains challenging, due to the highly promiscuous nature of TCR–pMHC binding and the resource-intensive demands of structural biology techniques [7, 8].

In recent years, AlphaFold [9–11] has made significant breakthroughs in modeling protein–peptide [12], antibody–antigen [13–15], and other complexes [16], offering immense potential for its application in TCR–pMHC complex modeling. Building on AlphaFold’s success, a growing number of methods have emerged [17–27]. These can be broadly categorized into **multiple sequence alignment (MSA)-based folding methods** that leverage co-evolutionary information from large genetic databases; **protein language model (PLM)-based folding methods** that learn evolutionary features directly from single sequences, enabling faster predictions for fast-evolving proteins [19]; and **docking-based methods**, which integrate deep learning-based folding models with physics-based docking algorithms. Recent approaches further incorporate acceleration strategies to meet the growing demand for high-throughput and rapid modeling [28]. However, existing studies often adopt inconsistent benchmarks and evaluation pipelines, which limit the fairness and practical applicability of current methods and make it difficult for practitioners to identify suitable tools.

Given the absence of consistent benchmarks for TCR–pMHC modeling, we systematically compare the three major categories of structure prediction methods (i.e. MSA-based, PLM-based, and docking-based) on 70 diverse and previously unseen TCR–pMHC complexes curated from TCR3d [29, 30]. The benchmark covers both class I (56 samples) and class II (14 samples) complexes. For each method, we evaluate docking quality using DockQ [31] and structural accuracy using root mean square deviation (RMSD) and TM-score [32]. Our evaluation reveals that MSA-based methods, particularly AlphaFold3, consistently outperform all alternatives across both docking and structural metrics, achieving a median DockQ score above 0.6 for both class I and class II complexes. In addition, AlphaFold3 replication models (Boltz-1 [22], Chai-1 [21], OpenFold3 [24], and Protenix-v1 [26]) achieved performance comparable with AlphaFold3. AlphaFold2 also remains competitive, and its efficient variants, TCRmodel2 [33] and ColabFold [28], achieve over 70-fold acceleration without compromising accuracy, enabling scalable high-throughput TCR–pMHC screening.

Building on the superior performance of AlphaFold3, we then explore its interpretability and practical utility by analyzing the predicted local distance difference test (pLDDT) score for the complementarity-determining region 3 (CDR3), which is a highly variable region that governs antigen-specific recognition and plays a decisive role in TCR–pMHC binding [34–37]. As a result, we find that AlphaFold3’s CDR3 pLDDT (CDR3 pLDDT denotes the average pLDDT score of all residues in the CDR3$\alpha$ and CDR3$\beta$ regions.) strongly correlates with both local modeling accuracy and overall docking quality. Leveraging this insight, we introduce a simple yet effective reranking strategy based on CDR3 pLDDT, which improves the Top-1 success rates of high- and medium-quality AlphaFold3 predictions by 2.9% and 4.3%, respectively. To further assess whether CDR3 pLDDT can aid AlphaFold3 in capturing the structure–function relationship, we model both wild-type and single-residue mutants and show that CDR3 pLDDT changes closely track mutation-induced binding affinity shifts, accurately reflecting functional alterations in 75.3% of cases. These results demonstrate that CDR3 pLDDT not only reflects structural reliability but also serves as a built-in signal that empowers AlphaFold3 to infer functionally meaningful changes in TCR–pMHC interactions.

In summary, we present a comprehensive and unified benchmark for TCR–pMHC complex modeling, covering diverse structure prediction paradigms and providing systematic evaluations of both accuracy and efficiency. Beyond the benchmark, we reveal the practical utility of AlphaFold3’s CDR3 pLDDT score for output selection and mutation effect prediction. Together, these contributions lay a scalable and interpretable foundation for TCR–pMHC modeling, with broad implications for immune repertoire analysis, therapeutic discovery, and personalized immunotherapy design.

## Materials and methods

### Data curation

#### Benchmark dataset for structure prediction evaluation

We constructed a nonredundant benchmark dataset of TCR–pMHC complexes to evaluate model performance on previously unseen structures. The dataset was curated from the TCR3d database [29, 30], which compiles experimentally resolved TCR–pMHC complexes from the Protein Data Bank (PDB) [38]. To ensure reliable benchmarking, we applied the following filtering criteria: (i) redundancy was removed to retain unique complexes; (ii) structures with a resolution higher than 3.5 Å were excluded to maintain model quality; and (iii) to avoid any overlap with training data used by evaluated models, only complexes released after 30 September 2021, the latest known training cutoff across all models, were included. For consistency in model inputs, each structure was preprocessed to retain only the TCR $\alpha$/$\beta$ variable domains, the peptide, and the peptide-binding domains of the MHC (i.e. $\alpha 1$/$\alpha 2$ for class I and $\alpha 1$/$\beta 1$ for class II). The benchmark dataset includes 56 class I and 14 class II complexes published between 30 September 2021 and 30 August 2024, with resolutions of 1.50–3.50 Å. It covers 51 unique TCRs, 22 distinct MHC molecules, and 53 unique peptides ranging from 8 to 15 amino acids, with 9-mer peptides being the most prevalent. Supplementary Fig. 14 provides detailed statistics.

To further reduce performance overestimation arising from sequence or structural similarity to the training data, we derived two stringent filtered subsets following established protocols [39]. First, we constructed a sequence-similarity-filtered subset using MMseqs2 [40, 41] to assess chain-wise homology. A complex was excluded if all of its chains showed >40% sequence identity to the corresponding chains of any single complex in the training set, resulting in a subset of 60 complexes. We then generated a structure-similarity-filtered subset using Foldseek-Multimer [42] by excluding any complex with a TM-score greater than 0.9 relative to the training set, yielding a more challenging subset of 30 complexes. The PDB IDs of the full benchmark set and both filtered subsets are listed in the Supplementary Information.

#### $\Delta \Delta G$ -oriented mutation dataset for confidence-affinity analysis

To explore the link between structural confidence and functional impact, we constructed two mutation datasets with binding free energy change ($\Delta \Delta G$) data. The first dataset, denoted as the $\Delta \Delta G$-SKEMPI set, was derived from the SKEMPI v2 database [43], which reports experimentally measured binding affinity changes in protein–protein complexes. Specifically, we retained wild-type and mutant pairs of TCR–pMHC complexes with single-point mutations exclusively within the TCR CDR3 region. After filtering for completeness and relevance, the final dataset comprised 21 unique mutations across three different TCR–pMHC complexes, each associated with an experimentally measured binding free energy change (Supplementary Table 5). Among them, eight mutations exhibited $\Delta \Delta G < 0$, indicating increased affinity in the mutant, while 13 showed $\Delta \Delta G> 0$, corresponding to reduced affinity.

To further mitigate potential overestimation caused by temporal or structural overlap with the training data, we constructed a $\Delta \Delta G$-FoldX set for a more stringent evaluation of model generalization. Specifically, we selected five TCR–pMHC complexes from our benchmark set that exhibited low structural similarity to the training set (TM-score < 0.85). For each complex, single-point mutations were introduced at key residues within the CDR3 region (positions 109–113 in the IMGT [44] numbering scheme). By sampling five random substitutions at each position, we generated 60 unique variants (Supplementary Table 6), and their $\Delta \Delta G$ values were computed using the FoldX [45] force field. The resulting dataset contained nine mutations with $\Delta \Delta G < 0$ and 51 with $\Delta \Delta G> 0$.

### Settings of benchmarked models

To ensure a fair and reproducible evaluation across major structure prediction paradigms, we standardized the execution settings of all 13 benchmarked models for TCR–pMHC structure prediction. For each model, only the top-ranked predicted structure for each complex, as determined by its internal ranking or scoring function, was retained for downstream analysis. The detailed model configurations are described below; unless otherwise specified, default parameters were used.

**AlphaFold2 [9].** We used the AlphaFold-Multimer [10] implementation from the official v2.3.2 release. MSAs were generated using HHBlits 3.3.0 [46] and Jackhmmer from HMMER 3.4 [47]. The PDB template database cutoff was set to 30 September 2021, and the random seed was fixed at 0. For each complex, five candidate structures were generated using the five available model parameter sets, and the top-ranked prediction was retained.

**AlphaFold3 [11].** Predictions were obtained from the AlphaFold3 public web server (https://alphafoldserver.com) using a random seed of 0. The PDB template database cutoff was restricted to 30 September 2021. For each TCR–pMHC complex, five candidate structures were generated, and the top-ranked prediction was retained.

**Boltz-1 [22].** We executed Boltz-1 with MSAs generated using the ColabFold MSA server (https://api.colabfold.com). The random seed was fixed at 0. Boltz-2 [23] was excluded from our benchmark because its training data cutoff date was 1 June 2023, which would introduce a risk of data leakage and partially confound the comparison.

**OpenFold3 [24].** We employed the OpenFold3-preview model. MSAs were generated using the ColabFold MSA server, with the use_template option enabled. The random seed was set to its default value of 42.

**Protenix-v1 [26].** We used the protenix_base_default_v1.0.0 model, which was trained with a data cutoff aligned with AlphaFold3. MSAs were generated using the Protenix MSA server (https://protenix-server.com/api/msa). The random seed was set to the default value of 101, and template information was enabled during prediction.

**RoseTTAFold2 [18].** The model was executed locally using the RF2_jan24 weights. MSAs were generated with HHblits 3.3.0 [46]. The random seed was fixed at 0.

**Chai-1-MSA [21].** Predictions were generated using the Chai Discovery public web server (https://lab.chaidiscovery.com) with the Use MSAs option enabled. In this configuration, MSAs are incorporated to improve prediction accuracy. The random seed was set to the default value of 42.

**Chai-1-PLM [21].** This model was executed on the Chai Discovery server (https://lab.chaidiscovery.com) with Use MSAs disabled, such that no MSAs were provided and evolutionary representations were derived solely from the pretrained PLM. The random seed was set to 42.

**ESM3 [48].** We used the 1.4B-parameter open-source checkpoint. The model was configured with num_steps = 3 and temperature = 0 to ensure stable structure generation from full complex sequences, with the random seed fixed at 0.

**ESMFold [20].** We used the esmfold_3B_v1 model with the random seed fixed at 0. Because ESMFold does not natively support multi-chain inputs, the TCR, peptide, and MHC sequences were concatenated using a 25-residue poly-glycine linker. This strategy approximates inter-chain flexibility while preserving compatibility with the single-chain model architecture.

**OmegaFold [19].** We used the model1 weights with num_cycles = 10 and a random seed of 0. As in ESMFold, multi-chain complexes were represented by concatenating the sequences with a 25-residue poly-glycine linker to facilitate structure prediction across the interface.

**AlphaRED [27].** We used AlphaFold2-predicted TCR–pMHC complexes as the initial configurations. The TCR and pMHC components were defined as the receptor and ligand, respectively. The interface pLDDT score was first calculated to determine whether global rigid-body docking was required. This was followed by flexible local refinement using ReplicaDock2 [49] with n_replica = 3. The final prediction was taken as the structure with the lowest interface energy score.

**AF2+HDOCK.** We employed the HDOCKlite v1.1 [50] standalone package (http://huanglab.phys.hust.edu.cn/software/hdocklite/) to perform template-based docking. Similar to AlphaRED, the AlphaFold2-predicted TCR and pMHC structures were submitted as separate inputs. The algorithm generated 20 docking poses per complex, and the candidate with the lowest energy score was selected for evaluation.

### Computational environment

All models were run locally, except for AlphaFold3 and Chai-1, which were accessed through web servers. The local hardware consisted of two Intel(R) Xeon(R) Gold 6348 CPUs (2.60 GHz) and an NVIDIA GeForce RTX 3090 GPU.

### Evaluation metrics

**Accuracy metrics.** To assess the accuracy of predicted TCR–pMHC structures, we employed RMSD, TM-score [32], and DockQ [31] as key evaluation metrics at the C$\alpha$ atom level. RMSD quantifies the average distance between corresponding atoms in the predicted and experimentally determined structures, serving as a standard indicator of structural deviation. Prior to RMSD calculation, the predicted and native structures were aligned using the Kabsch algorithm [51] to minimize positional differences across C$\alpha$ atoms. TM-score offers a scale-independent measure of structural similarity, overcoming RMSD’s sensitivity to outliers and dependency on protein size. TM-score values range from 0 to 1, where higher scores indicate greater structural similarity, with values above 0.5 generally suggesting a correct overall fold [52]. We used both RMSD and TM-score to comprehensively assess modeling accuracy for the entire TCR–pMHC complex and its specific regions. DockQ is a continuous metric for measuring docking model quality, combining three components: $\mathrm{F}_{\mathrm{nat}}$ (the fraction of native interfacial contacts preserved in the predicted complex), LRMS (the ligand RMSD after superimposing the receptor), and iRMS (the interface RMSD between predicted and native interfacial residues). DockQ scores range from 0 to 1 and are calibrated against the Critical Assessment of Predicted Interactions (CAPRI) criteria [53, 54], classifying docking models into four categories: incorrect ($\mathrm{DockQ}<0.23$), acceptable ($0.23\le \mathrm{DockQ}<0.49$), medium quality ($0.49\le \mathrm{DockQ}<0.80$), and high quality ($\mathrm{DockQ}\ge 0.80$).

**Confidence metrics.** The pLDDT is a residue-level confidence metric proposed by AlphaFold2 [9] that quantifies prediction reliability. For specific regions of interest, such as the complementarity-determining region 3 (CDR3) loop of the TCR $\alpha$ and $\beta$ chains, the regional pLDDT score was calculated as the average pLDDT of all residues within the region.

## Results

### Benchmarking structure prediction methods for TCR–pMHC complexes

**Figure 1:**
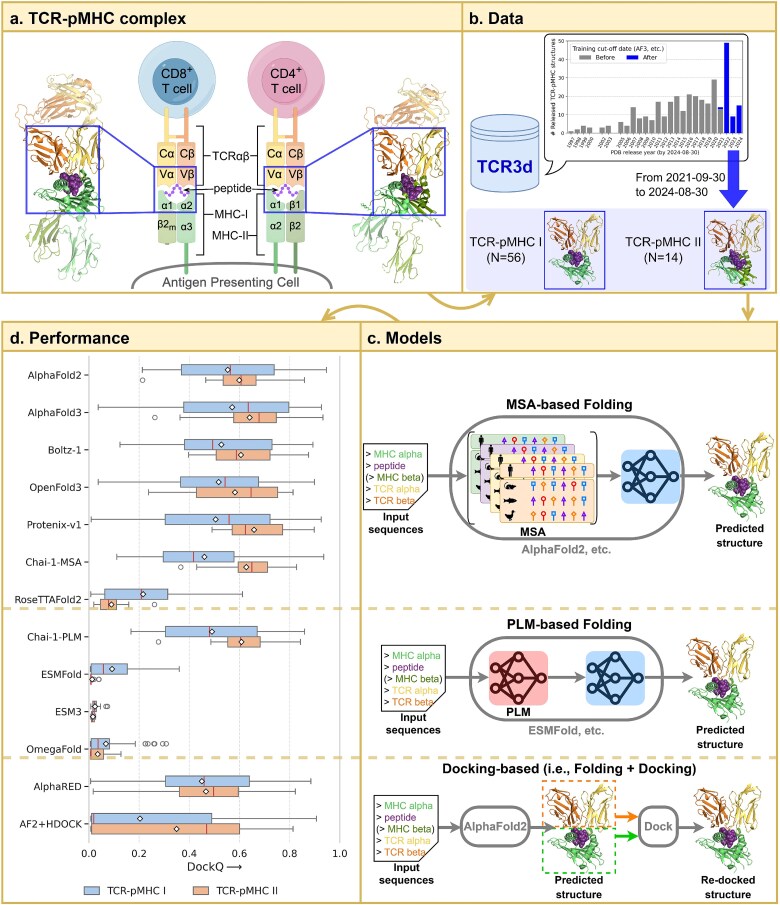
**Overview of the benchmark for TCR–pMHC complex modeling. a.** Schematic of a TCR–pMHC interaction. Key regions include the variable domains of the TCR $\alpha$ and $\beta$ chains, the peptide, and the peptide-binding regions of class I ($\alpha$1 and $\alpha$2) or class II ($\alpha$1 and $\beta$1) MHC. **b.** Benchmark dataset comprising 56 class I and 14 class II TCR–pMHC structures from the TCR3d database. All 70 structures were released after the AlphaFold3 training cutoff date (30 September 2021), ensuring they were unseen during model training. **c.** Overview of three evaluated structure prediction paradigms. MSA-based methods use MLMs to guide structure prediction via co-evolutionary signals. PLM-based methods replace MSA with PLMs for single-sequence inference. Docking-based methods refine predicted structures (e.g. from AlphaFold2) using docking algorithms to reorient the TCR and pMHC. **d.** Box-plot comparison of Top-1 DockQ scores across 13 representative models on the benchmark dataset. In each box plot, the horizontal line indicates the median, and the diamond marker indicates the mean. AlphaFold3 achieved the highest median DockQ scores for both class I (0.636) and class II (0.679) TCR–pMHC complexes. In terms of mean DockQ scores, AlphaFold3 ranked the highest for class I complexes (0.571), whereas Protenix-v1 performed the best for class II complexes (0.658).

We began by benchmarking representative structure prediction models on a curated set of unseen TCR–pMHC complexes. Three categories of models (Fig. 1c) were evaluated: MSA-based folding models (AlphaFold2 [9], AlphaFold3 [11], Boltz-1 [22], OpenFold3 [24], Protenix-v1 [26], Chai-1-MSA [21], RoseTTAFold2 [18]), PLM-based folding models (Chai-1-PLM [21], ESM3 [48], ESMFold [20], OmegaFold [19]), and docking-based approaches (AlphaRED [27] and HDOCK [50] applied to AlphaFold2 outputs). Our evaluations focused on the primary interacting regions of the complexes, including the variable domains of the TCR $\alpha$ and $\beta$ chains, the peptide, and the peptide-binding domains of the MHC (Fig. 1a). The benchmark comprised 70 nonredundant structures (56 class I and 14 class II) from the TCR3d database (Fig. 1b), all released after 30 September 2021, ensuring they were excluded from the training data and structural templates of all evaluated models.

**Figure 2:**
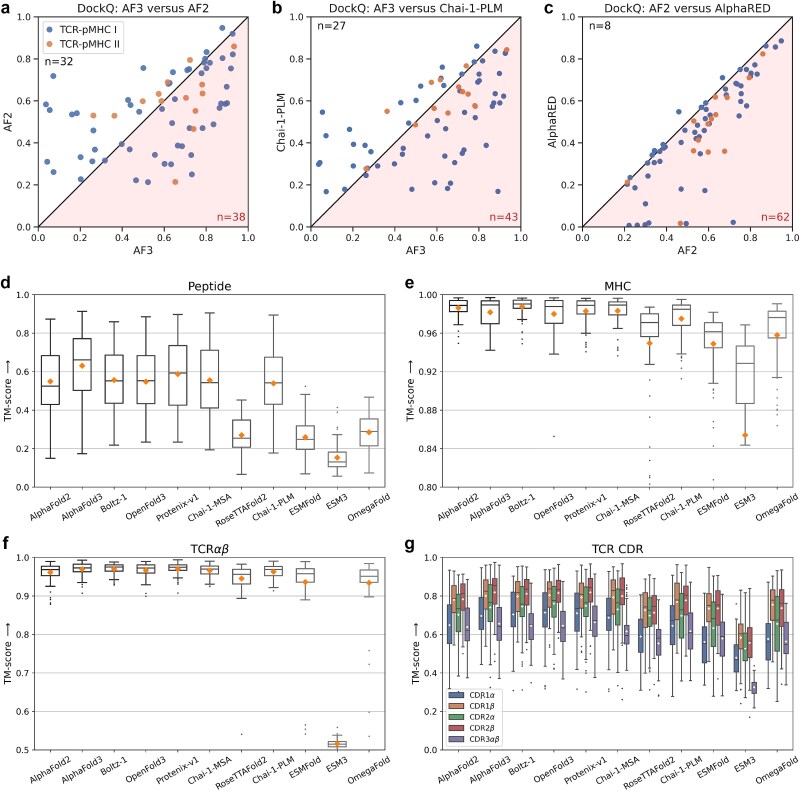
**Detailed comparison of benchmark results. a–c.** DockQ scatter plots comparing Top-1 predicted structures across different models: **a.** AlphaFold3 versus AlphaFold2, **b.** AlphaFold3 versus the best PLM-based model, and **c.** AlphaFold2 versus AlphaRED. **d–g.** TM-score distributions of Top-1 predicted structures by MSA-based and PLM-based models for **d.** peptide, **e.** MHC, **f.** TCR, and **g.** TCR CDR loops. In each box plot, the horizontal line indicates the median, and the diamond marker indicates the mean.

**Superior performance of AlphaFold3.** DockQ-based evaluation of overall structural docking quality is summarized in Fig. 1d. AlphaFold3 achieved the highest median DockQ scores across both class I (0.636) and class II (0.679) complexes, indicating that the majority of its predicted structures are of medium to high quality. Component-level modeling accuracy evaluations further revealed that AlphaFold3 generated highly accurate models for the TCR, peptide, and MHC regions, with median and mean TM-scores exceeding 0.6 (Fig. 2d–g, Supplementary Tables 1 and 2). After removing data with high sequence and structural similarity to the training set (see Materials and methods), AlphaFold3 still maintained superior performance (Supplementary Figs 5, 6, and 7). Through web access, AlphaFold3 could deliver results for each sample within 2 min (Supplementary Table 3), which is suitable for routine usage.

**Performance of other MSA-based methods.** AlphaFold3 replication models, including Boltz-1, Chai-1-MSA, OpenFold3, and Protenix-v1, achieved comparable overall performance (Fig. 1d, Supplementary Fig. 5), accurately modeling individual chains but with some gaps in peptide modeling compared with AlphaFold3 (Fig. 2d–g, Supplementary Tables 1 and 2). Boltz-1 showed the most similar performance to AlphaFold3 (Supplementary Fig. 1a–d) and had a comparable local runtime with AlphaFold3’s web server (115.3 seconds per sample, Supplementary Table 3). Protenix-v1 achieved higher mean DockQ scores than AlphaFold3 on class II structures, while performing slightly worse on class I structures (Fig. 1d, Supplementary Fig. 5). Chai-1-MSA performed comparably with AlphaFold3 on class II complexes, but underperformed on class I complexes (Fig. 1d). These results support the effectiveness of the AlphaFold3 methodological framework for achieving superior performance in TCR–pMHC modeling.

AlphaFold2 showed slightly lower DockQ performance compared with AlphaFold3, and offered complementary predictions (Fig. 2a). In particular, AlphaFold2 occasionally generated medium-quality predictions (DockQ > 0.49) for complexes that AlphaFold3 failed to model correctly (DockQ < 0.23). Additionally, AlphaFold2 demonstrated more stable performance in MHC modeling, exhibiting lower variance than AlphaFold3 (Fig. 2e). RoseTTAFold2 exhibited consistently lower performance than other MSA-based models, with reduced docking quality and notably poor peptide modeling (Fig. 2d).

While AlphaFold2 remained competitive in DockQ and structural accuracy, its routine deployment was hampered by the computationally intensive MSA generation step (taking over 120 min per TCR–pMHC complex in our local setup). We further evaluated two MSA-acceleration strategies based on AlphaFold2: TCRmodel2 [33] with a curated TCR/MHC sequence database, and ColabFold [28] with MMseqs2 [40, 41] for rapid homology search. Both approaches yielded similar DockQ and TM-score distributions to the original AlphaFold2 (Supplementary Fig. 2a,b), while reducing MSA runtime per complex by over 70-fold (from 123.5 min to $\sim$1.7 min, Supplementary Fig. 2c). This highlights the practicality of AlphaFold2 when combined with optimized MSA strategies, enabling accurate and scalable TCR–pMHC structure prediction in resource-constrained settings.

**Performance of PLM-based methods.** In contrast, most PLM-based models underperformed relative to MSA-based models. ESMFold, OmegaFold, and ESM3 generally failed to produce accurate complex structures, despite their faster inference (Supplementary Table 3). For ESMFold and OmegaFold, the main limitation was their lack of multi-chain modeling support: both models treat multi-chain systems as single-chain inputs, resulting in incorrect inter-chain orientations. These two models produced reasonably accurate predictions for individual components such as TCR and CDR loops (Fig. 2f,g), but their peptide modeling was notably poor (Fig. 2d). In the case of ESM3, performance was further limited by its reliance on generative modeling and the use of its smallest publicly available checkpoint. Notably, Chai-1-PLM emerged as an exception among PLM models. It achieved performance comparable with Chai-1-MSA (Supplementary Figs 1e, 6f, and 7f) and approached AlphaFold-level quality, even outperforming AlphaFold3 in over one-third of the tested complexes (Fig. 2b). This can be attributed to Chai-1’s AlphaFold3-style architecture, which incorporates chain-aware adaptations and jointly leverages MSA and PLM features during training.

**Performance of docking-based methods.** Finally, docking-based methods failed to enhance the docking quality of the AlphaFold2-generated TCR–pMHC complexes, despite requiring additional docking time (Supplementary Table 3). In AlphaRED, we performed rigid-body docking between separately predicted TCR and pMHC structures, followed by flexible refinement guided by interface pLDDT scores. However, this approach improved DockQ scores in only 8 of the 70 complexes, while performance declined in the remaining cases (Fig. 2c). A similar outcome was observed with HDOCK (Supplementary Fig. 1f), suggesting that current docking strategies offer limited added value when starting from high-quality end-to-end predictions.

**Modeling performance of pMHC within TCR–pMHC.** Accurate reconstruction of the peptide–MHC (pMHC) sub-complex is critical for reliable TCR docking [55]. We therefore assessed the performance of folding models in modeling the pMHC sub-complex within full TCR–pMHC complexes. As shown in Supplementary Fig. 12, AlphaFold3 achieved the strongest overall performance. Both AlphaFold3 replication models and AlphaFold2 yielded high-quality pMHC predictions, with median and mean DockQ scores both above 0.8. Across these models, pMHC DockQ remained uniformly high, whereas the overall TCR–pMHC DockQ scores varied widely, spanning the full range from 0 to 1 (Supplementary Fig. 13). These results suggest that the main bottleneck in TCR–pMHC structure prediction lies not in modeling peptide positioning within the MHC groove, but in resolving the geometric complexity of TCR docking orientation [56].

### CDR3 pLDDT: a key modeling and functional indicator for AlphaFold3

AlphaFold3 provides well-calibrated confidence scores aligned with structural accuracy and docking quality [11], serving as valuable internal indicators of model reliability. Among these, the pLDDT score serves as a fine-grained metric of local confidence. Given the central role of the CDR3 loops of TCR $\alpha$ and $\beta$ chains in antigen recognition and their inherent sequence variability [57, 58], we investigated whether the CDR3 pLDDT score could provide deeper insights into modeling quality and functional relevance. Our analysis shows that CDR3 pLDDT is not only indicative of local modeling accuracy and global docking quality, but also useful for reranking multiple model outputs and detecting affinity-altering mutations.

#### CDR3 pLDDT reflects CDR3 modeling accuracy and docking quality

**Figure 3:**
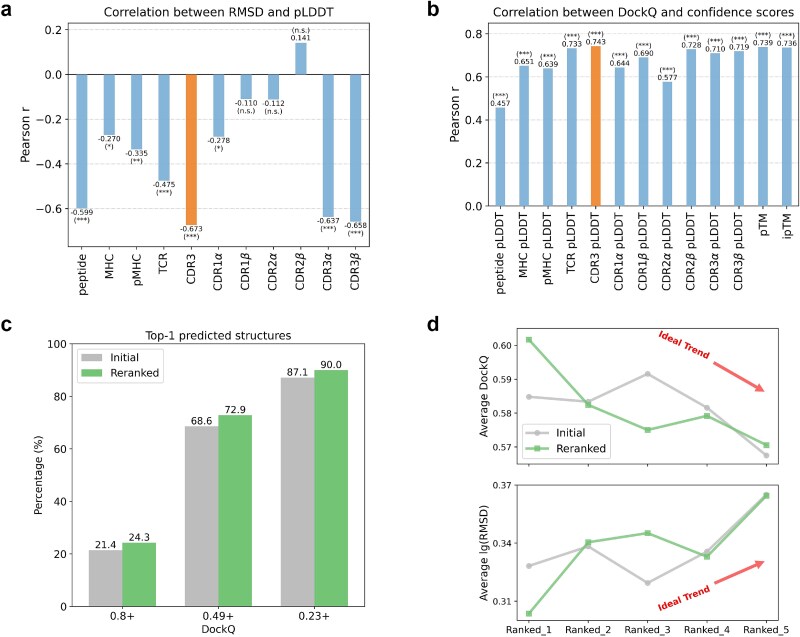
**The role of CDR3 pLDDT in predicted TCR–pMHC complexes by AlphaFold3. a.** Correlation between pLDDT scores and RMSD across different regions. CDR3 pLDDT shows the strongest correlation with CDR3 RMSD. Statistical significance is indicated as ^*^$\textit{P}<\mathrm{0.05}$; ^*^^*^$\textit{P}<\mathrm{0.01}$; ^*^^*^^*^$\textit{P}<\mathrm{0.001}$; n.s., not significant. **b.** Correlation between confidence scores (including pLDDT scores for different regions and pTM/ipTM) and DockQ, where CDR3 pLDDT shows the strongest correlation. **c.** Comparison of DockQ distributions for Top-1 predicted structures before and after reranking AlphaFold3’s five predicted results using CDR3 pLDDT. Reranking improves the overall docking quality of Top-1 predictions. **d.** CDR3 pLDDT-based reranking enhances consistency between prediction accuracy and ranking, ensuring better alignment of docking quality with structure rankings.

We first analyzed the relationship between per-residue confidence (pLDDT) and structural accuracy (RMSD) for different regions of the complex. As shown in Fig. 3a, the correlation between pLDDT and RMSD for CDR3 was found to be the most significant, with a Pearson correlation coefficient of −0.673, indicating that CDR3 pLDDT precisely reflects the accuracy of CDR3 modeling. Meanwhile, we examined the relationship of CDR3 pLDDT and docking quality (DockQ). CDR3 pLDDT correlated more strongly with DockQ than pLDDT scores from other regions of the complex or ipTM/pTM scores, which assess interface/global model quality in AlphaFold (Fig. 3b). CDR3$\alpha$ pLDDT and CDR3$\beta$ pLDDT exhibited similar correlations with regional RMSD (−0.637 versus −0.658 in Fig. 3a) and DockQ (0.710 versus 0.719 in Fig. 3b), but neither correlated as strongly as the unified CDR3 pLDDT. In addition, the correlation between CDR3 pLDDT and DockQ/regional RMSD remained the strongest when considering class I and class II TCR–pMHC complexes separately (Supplementary Figs 9 and 10). This highlights that CDR3 pLDDT alone provides a robust local proxy for overall docking accuracy.

#### CDR3 pLDDT-based reranking enhances Top-1 structure prediction

Given the strong correlation between CDR3 pLDDT and docking quality, we reranked AlphaFold3’s five predictions using CDR3 pLDDT instead of the original ranking score. This reranking strategy effectively corrected errors introduced by the initial ranking. For the Top-1 predicted structure (Fig. 3c), reranking based on CDR3 pLDDT increased the success rate of high and medium docking quality predictions by 2.9% and 4.3%, respectively, effectively preventing the omission of high-quality predicted structures. Further analysis of DockQ changes after reranking showed that the effect was minimal for most samples. In contrast, two samples, 7N2O and 7DZN, which had lower structural similarity to the training set (TM-score < 0.85), exhibited DockQ gains greater than 0.3 (Supplementary Fig. 11). In contrast, reranking using ipTM, pTM, AlphaFold2’s ranking score, or other regional pLDDT scores yielded less improvement than CDR3 pLDDT-based reranking (Supplementary Fig. 4a). Notably, increasing the number of predictions for reranking consistently improves the reranked Top-1 quality (Supplementary Fig. 4b). Moreover, after reranking all five predictions for each complex, the docking quality and modeling accuracy showed greater consistency with the ranking (Fig. 3d), improving the reliability and confidence of the predictions. However, applying the same CDR3 pLDDT-based reranking to AlphaFold2 yielded limited improvement, with a lower correlation ($\sim$0.4) between CDR3 pLDDT and DockQ (Supplementary Fig. 3). This may be due to AlphaFold2’s earlier confidence head, which can produce unreliable pLDDT values [59], in contrast to the refined confidence head in AlphaFold3.

#### CDR3 pLDDT reflects affinity changes induced by CDR3 mutations

**Figure 4:**
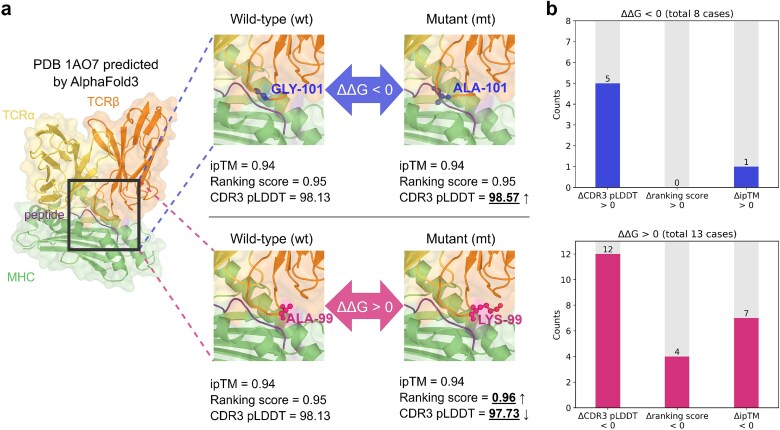
**The role of CDR3 pLDDT in mutation-induced TCR–pMHC binding affinity changes. a.** Comparison of confidence metrics for two CDR3 mutations in the 1AO7 complex with one positive and one negative $\Delta \Delta G$. **b.** Consistency between confidence score changes and binding affinity changes across 21 CDR3 mutations. CDR3 pLDDT changes align with the direction of affinity change in 62.5% (5/8) of $\Delta \Delta G < 0$ cases with increased affinity and 92.3% (12/13) of $\Delta \Delta G> 0$ cases with decreased affinity, outperforming ranking score and ipTM. $\Delta \Delta G$ is defined as $\Delta \Delta G = \Delta G_{\mathrm{mutant}} - \Delta G_{\mathrm{wild-type}}$. A negative $\Delta \Delta G$ indicates that the mutation increases binding affinity, whereas a positive $\Delta \Delta G$ suggests a decrease in binding affinity.

To further explore the functional relevance of CDR3 modeling confidence, we investigated whether CDR3 pLDDT is sensitive to binding affinity changes caused by CDR3 mutations. We used AlphaFold3 to predict the structures of both wild-type and mutant variants from the $\Delta \Delta G$-SKEMPI dataset, which was derived from SKEMPI v2 [43]. For each prediction, we extracted the CDR3 pLDDT score, as well as AlphaFold3’s key confidence metrics, including ranking score and interface-focused ipTM, to assess their sensitivity to affinity-altering mutations. As shown in Fig. 4a, we compared the change in confidence scores between wild-type and mutant structures for two mutations from the 1AO7 complex, one associated with increased binding affinity ($\Delta \Delta G < 0$) and the other with decreased affinity ($\Delta \Delta G> 0$). As a result, CDR3 pLDDT consistently increased or decreased in accordance with the direction of affinity change, whereas the ranking score and ipTM either stayed constant or fluctuated in ways unrelated to the affinity shift. This observation was further validated across the full mutation dataset, where CDR3 pLDDT consistently aligned with the direction of binding affinity changes more reliably than AlphaFold3’s ranking score or ipTM (Fig. 4b). Specifically, five out of eight mutations associated with increased affinity ($\Delta \Delta G < 0$) exhibited an increase in CDR3 pLDDT; 12 out of the 13 mutations associated with decreased affinity ($\Delta \Delta G> 0$) showed a reduction in CDR3 pLDDT.

To further assess the robustness of this relationship under a setting less susceptible to potential temporal or structural biases, we extended our analysis to the $\Delta \Delta G$-FoldX set, focusing on complexes with low structural similarity to training data. In the representative 7N2O complex, CDR3 pLDDT again correctly reflected the direction of $\Delta \Delta G$ changes, whereas other confidence metrics failed to show clear discriminative ability (Supplementary Fig. 8a). Across the dataset, CDR3 pLDDT correctly identified the direction of affinity changes in five of nine enhancing mutations and 39 of 51 reducing mutations (Supplementary Fig. 8b). By integrating the results from both the $\Delta \Delta G$-SKEMPI and $\Delta \Delta G$-FoldX sets, CDR3 pLDDT successfully captured the direction of affinity changes in mutations in 75.3% (61/81) of cases. Despite the numerical imbalance between affinity-enhancing and affinity-reducing mutation sets, these preliminary findings suggest that CDR3 pLDDT holds potential as a qualitative indicator for structural changes associated with affinity-altering mutations.

## Conclusion and discussion

Using a curated set of 70 nonredundant TCR–pMHC complexes, we conducted a detailed evaluation of three prominent structure prediction methods for TCR–pMHC complex modeling, with AlphaFold3 [11] emerging as a robust performer. Our findings validated that MSA generation, a critical step in the AlphaFold pipeline, can be significantly accelerated by customizing the database [33] and employing MMseqs2 [40, 41] without loss of accuracy. This acceleration is crucial for enhancing the efficiency of AlphaFold, especially in large-scale applications where quick turnaround times are needed. Furthermore, our results demonstrate that CDR3 pLDDT serves as an effective structural confidence metric for reranking predictions, thereby improving the selection of near-native poses. While not a direct affinity predictor, this metric could sensitively reflect affinity changes induced by single-point mutations. In addition, the classification performance of CDR3 pLDDT on TCR–pMHC sequence datasets with large numbers of positive and negative samples was limited but detectable (Supplementary Fig. 15 and Supplementary Table 7). These findings highlight the value of biologically grounded insights in improving both the accuracy and interpretability of immune modeling tasks, and suggest that combining localized, biologically informed metrics such as CDR3 pLDDT with specialized binding predictors may improve the reliability of non-binder filtering.

Several directions could further improve and scale *in silico* TCR–pMHC modeling. Due to the limited availability of class II data, the statistical robustness of our benchmark will improve as additional class II structures become available. As structural geometric accuracy is the common evaluation criterion for current benchmarks, biologically meaningful metrics will further enhance benchmark utility. Although our results suggest that naive re-docking of predicted structures does not consistently improve docking quality, incorporating residue-level interaction information as restraints may yield better results. When integrated into docking algorithms such as HADDOCK [60], these constraints could guide interface modeling using biologically informed evidence. In parallel, the potential of PLMs [61] for structure prediction warrants further study. Our evaluation shows that Chai-1 [21] in single-sequence mode (Chai-1-PLM) performs comparably with AlphaFold in modeling TCR–pMHC complexes, highlighting the promise of PLM-based folding models for fast and accurate structure prediction. This may enable multimer-aware PLM-based models to support high-throughput neoantigen screening and accelerate the discovery of novel TCR targets. Moreover, although current evidence is still limited by the small number of mutation cases and the reliance on estimation-derived data, the observed association between CDR3 pLDDT and binding affinity shifts suggests that localized confidence metrics may complement global scores and support mutation-based TCR design through candidate prioritization [62–66]. Overall, integrating domain knowledge, advancing PLM-based modeling, and strengthening collaboration between computational and experimental studies will further expand the potential of in silico structural modeling in immunotherapy.

**Key Points**

- We provide a unified benchmark comparing MSA-based, PLM-based, and docking-based methods for TCR–pMHC structure prediction across 70 unseen complexes.
- MSA-based methods, particularly AlphaFold3, outperform PLM-based and docking-based approaches in modeling TCR–pMHC complex structures.
- CDR3 pLDDT serves as a robust indicator of structural reliability and reflects mutation-induced affinity changes in predicted TCR–pMHC structures.

## Supplementary Material

supp_bbag289

## Data Availability

The benchmark dataset is collected from https://tcr3d.ibbr.umd.edu/. All structures are available in PDB (https://www.rcsb.org/). The $\Delta \Delta G$-SKEMPI dataset is collected from https://life.bsc.es/pid/skempi2/. Our code is available at https://github.com/Jiadong001/TCR-pMHC-folding-benchmark. The codes for different benchmark models are available via GitHub at https://github.com/google-deepmind/alphafold, https://github.com/jwohlwend/boltz, https://github.com/bytedance/Protenix, https://github.com/aqlaboratory/openfold-3, https://github.com/uw-ipd/RoseTTAFold2, https://github.com/facebookresearch/esm, https://github.com/HeliXonProtein/OmegaFold, https://github.com/evolutionaryscale/esm, https://github.com/Graylab/AlphaRED, https://github.com/piercelab/tcrmodel2, and https://github.com/sokrypton/ColabFold.
