# Supplementary material for: Benchmarking TCR–pMHC structure prediction: a unified evaluation and CDR3-based functional insights
Source: Brief Bioinform. 2026 Jun 5;27(3):bbag289. doi: 10.1093/bib/bbag289 (PMC13240595; doi:10.1093/bib/bbag289)
Supplement: supp_bbag289 [file supp_bbag289.pdf]

Benchmarking TCR–pMHC structure prediction: a unified evaluation and CDR3-based functional insights

Supplementary Information

1 PDB IDs

All PDB IDs used in the benchmark: 6ZKW, 6ZKX, 6ZKY, 6ZKZ, 7DZM, 7DZN, 7L1D, 7N2N, 7N2O, 7N2P, 7N2Q, 7N2R, 7N2S, 7N4K, 7N5C, 7N5P, 7NA5, 7NDQ, 7NDT, 7NDU, 7NME, 7NMF, 7NMG, 7OW5, 7OW6, 7PB2, 7PBE, 7PHR, 7Q99, 7Q9A, 7Q9B, 7QPJ, 7R80, 7RK7, 7RM4, 7RRG, 7RTR, 7SU9, 8CX4, 8D5Q, 8DNT, 8EN8, 8ENH, 8EO8, 8F5A, 8GOM, 8GON, 8GVB, 8GVG, 8GVI, 8I5C, 8I5D, 8QFY, 8SHI, 8WTE, 8WUL, 7RDV, 7SG0, 7SG1, 7SG2, 7T2B, 7T2C, 7T2D, 7Z50, 8PJG, 8TRL, 8TRR, 8VCX, 8VCY, 8VD2.

PDB IDs after sequence-similarity filtering: 6ZKW, 6ZKX, 6ZKY, 6ZKZ, 7DZM, 7DZN, 7L1D, 7N2N, 7N2O, 7N2P, 7N2Q, 7N2R, 7N2S, 7N5C, 7N5P, 7NA5, 7NDQ, 7NDT, 7NDU, 7NME, 7NMF, 7NMG, 7OW5, 7OW6, 7PB2, 7Q99, 7Q9A, 7QPJ, 7R80, 7RK7, 7RRG, 7SU9, 8CX4, 8D5Q, 8DNT, 8EN8, 8ENH, 8EO8, 8F5A, 8GON, 8GVB, 8GVG, 8GVI, 8I5C, 8I5D, 8QFY, 8SHI, 8WTE, 8WUL, 7RDV, 7SG2, 7T2B, 7T2C, 7T2D, 7Z50, 8TRL, 8TRR, 8VCX, 8VCY, 8VD2.

PDB IDs after structure-similarity filtering: 6ZKW, 6ZKX, 6ZKY, 6ZKZ, 7DZM, 7DZN, 7NA5, 7NDQ, 7NDU, 7OW5, 7OW6, 7PB2, 7RK7, 7RM4, 7RRG, 8D5Q, 8EN8, 8ENH, 8EO8, 8F5A, 8QFY, 8SHI, 8WTE, 8WUL, 7SG0, 7T2B, 7T2C, 7T2D, 8PJG, 8TRR.

2 Supplementary Figures

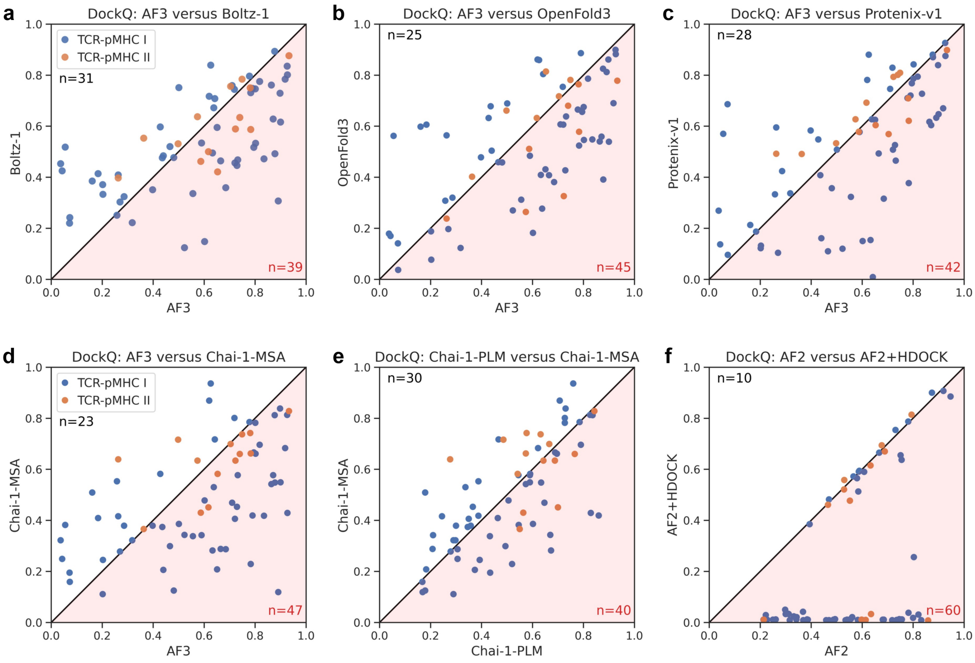

Supplementary Fig. 1 DockQ scatter plots comparing Top-1 predicted structures across different models. a. AlphaFold3 versus Boltz-1, **b.** AlphaFold3 versus OpenFold3, **c.** AlphaFold3 versus Protenix-v1, **d.** AlphaFold3 versus Chai-1-MSA, **e.** Chai-1-MSA versus Chai-1-PLM, and **f.** AlphaFold2 versus AlphaFold2+HDOCK.

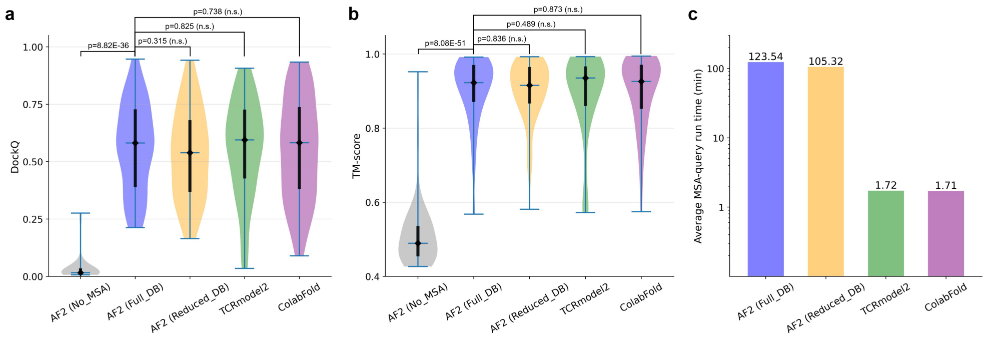

Supplementary Fig. 2 Impact of Accelerated MSA Strategies on AlphaFold2 Structure Prediction. We evaluate four MSA generation strategies for AlphaFold2: (1) AF2 (Full_DB): full database search with jackhmmer and HHblits, (2) AF2 (Reduced_DB): reduced database, (3) TCRmodel2 [1]: domain-specific database, and (4) ColabFold [2]: ColabFold database queried via MMseqs2 [3, 4]. **a-b.** Comparison of DockQ **(a)** and TM-score **(b)** distributions over all the 70 TCR–pMHC complexes. Naively omitting MSAs causes a significant drop in prediction accuracy. **c.** Average MSA search time per method. Both TCRmodel2 and ColabFold pipelines offer **over 70×** **acceleration** in MSA generation (reducing average search time to ∼1.7 minutes) compared to the full database (over 100 minutes), with negligible performance degradation.

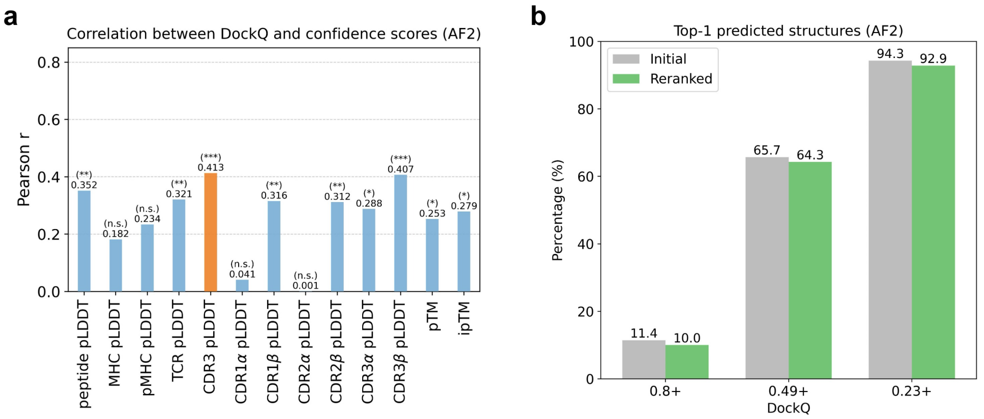

Supplementary Fig. 3 CDR3 pLDDT-based analysis for AlphaFold2. a. Correlation between confidence scores and DockQ. All correlation coefficients are below 0.5. CDR3 pLDDT shows the best correlation. **b.** Comparison of DockQ distributions for Top-1 predicted structures before and after reranking AlphaFold2's five predicted results using CDR3 pLDDT. Reranking with CDR3 pLDDT maintains the overall docking quality of the Top-1 predicted structures.

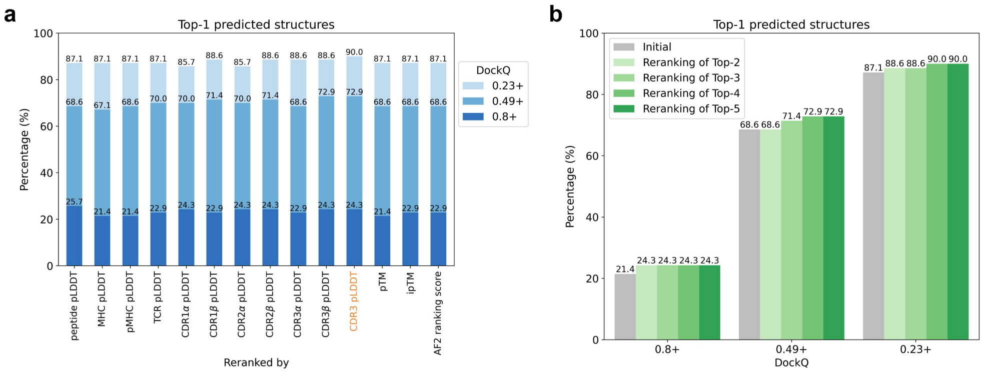

Supplementary Fig. 4 CDR3 pLDDT-based reranking improves docking quality of Top-1 predicted structures. a. Comparison of DockQ distributions of Top-1 predictions after reranking AlphaFold3 outputs using different confidence scores. CDR3 pLDDT-based reranking yields the greatest improvement. **b.** DockQ distributions of Top-1 predictions after reranking the top 1–5 AlphaFold3 outputs using CDR3 pLDDT. Performance improves with a larger reranking scope.

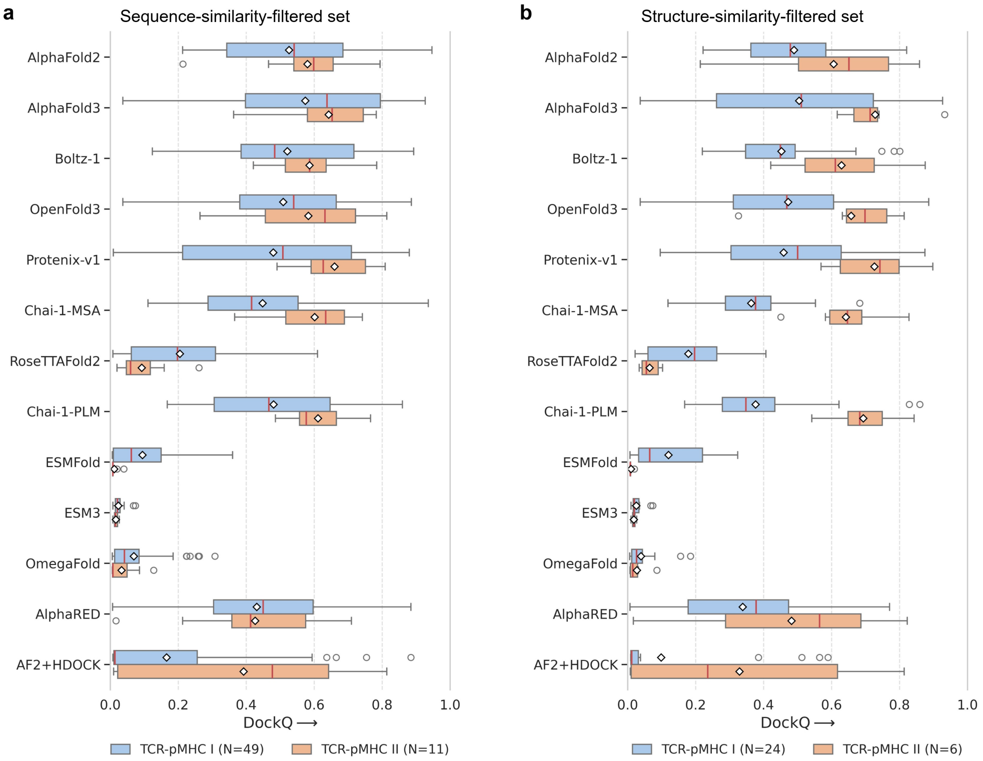

Supplementary Fig. 5 Box-plot comparison of Top-1 DockQ scores across 13 structure prediction models on the benchmark dataset filtered by similarity. a. Sequence-similarity based filtering. AlphaFold3 achieved the highest median DockQ scores: 0.638 for class I and 0.653 for class II TCR–pMHC complexes. For the highest mean DockQ scores, AlphaFold3 achieved 0.574 for class I, and Protenix-v1 achieved 0.660 for class II. **b.** Structure-similarity based filtering. AlphaFold3 achieved the highest mean DockQ scores: 0.505 for class I and 0.728 for class II TCR–pMHC complexes. For the highest median DockQ scores, AlphaFold3 achieved 0.511 for class I, and Protenix-v1 achieved 0.742 for class II. Note that the line within each box denotes the median, and the diamond marker denotes the mean.

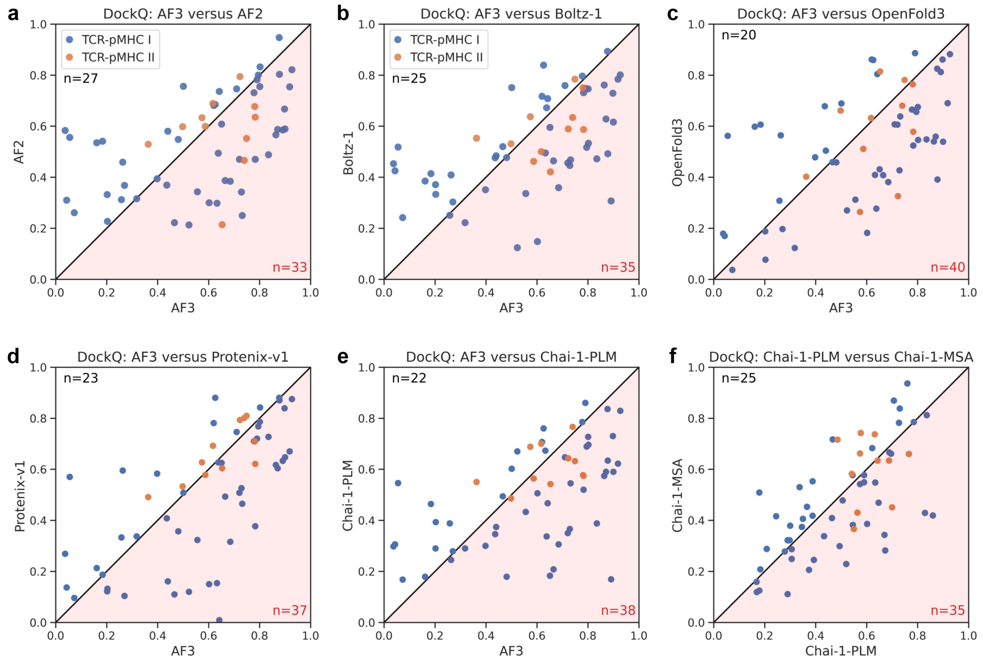

Supplementary Fig. 6 Head-to-head comparison between different models on sequence-similarity-filtered data. **a.** AlphaFold3 versus AlphaFold2, **b.** AlphaFold3 versus Boltz-1, **c.** AlphaFold3 versus OpenFold3, **d.** AlphaFold3 versus Protenix-v1, **e.** AlphaFold3 versus Chai-1-PLM, and **f.** Chai-1-MSA versus Chai-1-PLM.

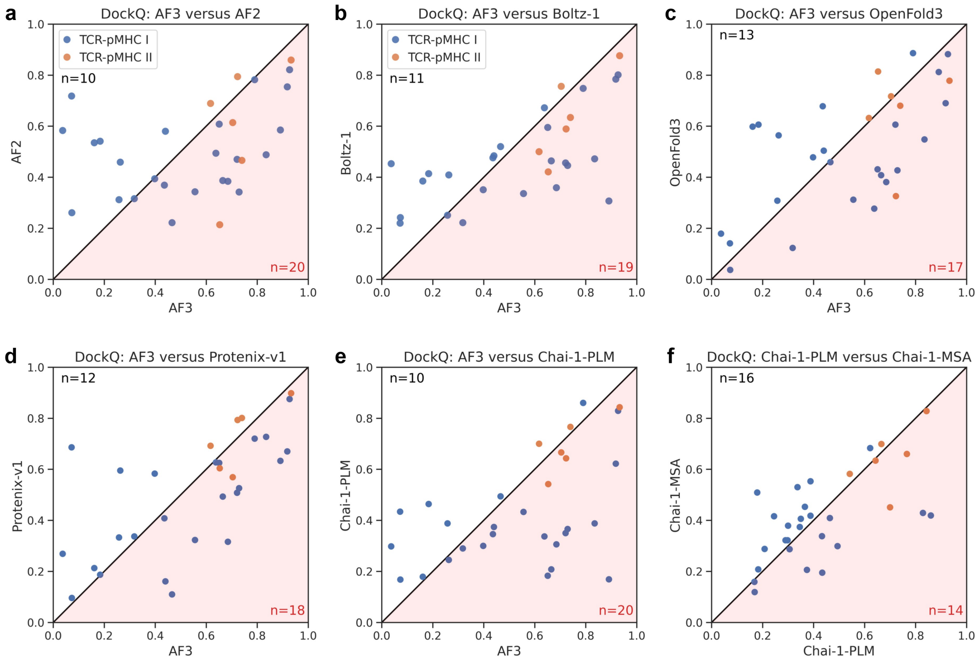

Supplementary Fig. 7 Head-to-head comparison between different models on structure-similarity-filtered data. **a.** AlphaFold3 versus AlphaFold2, **b.** AlphaFold3 versus Boltz-1, **c.** AlphaFold3 versus OpenFold3, **d.** AlphaFold3 versus Protenix-v1, **e.** AlphaFold3 versus Chai-1-PLM, and **f.** Chai-1-MSA versus Chai-1-PLM.

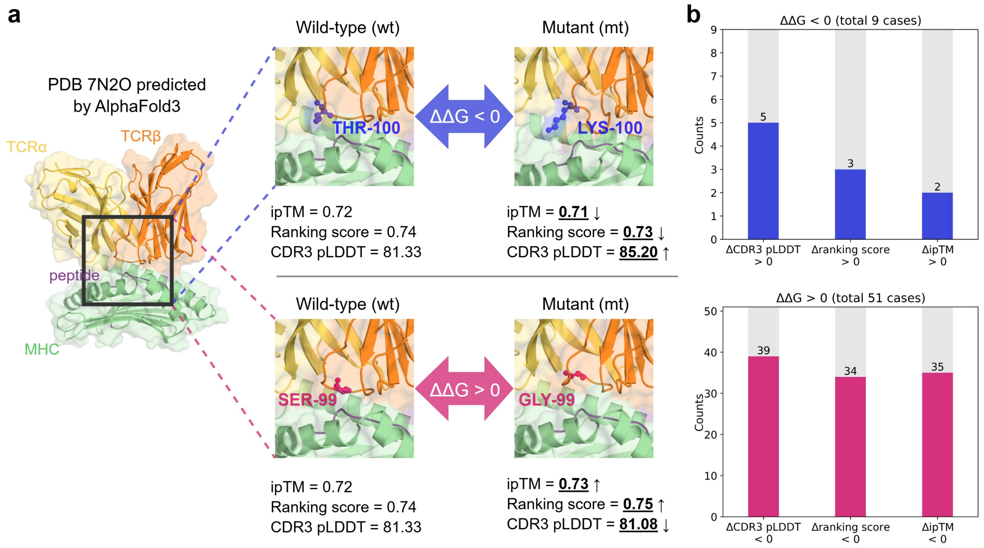

Supplementary Fig. 8 CDR3 pLDDT captures mutation-induced TCR–pMHC binding affinity changes on $\Delta\Delta G$-FoldX set. a. Comparison of confidence metrics for two CDR3 mutations in the 7NO2 complex with one positive and one negative $\Delta\Delta G$. **b.** Consistency between confidence score changes and binding affinity changes across 60 CDR3 mutations. CDR3 pLDDT changes align with the direction of affinity change in 55.6% (5/9) of $\Delta\Delta G < 0$ cases with increased affinity and 76.5% (39/51) of $\Delta\Delta G > 0$ cases with decreased affinity, outperforming ranking score and ipTM.

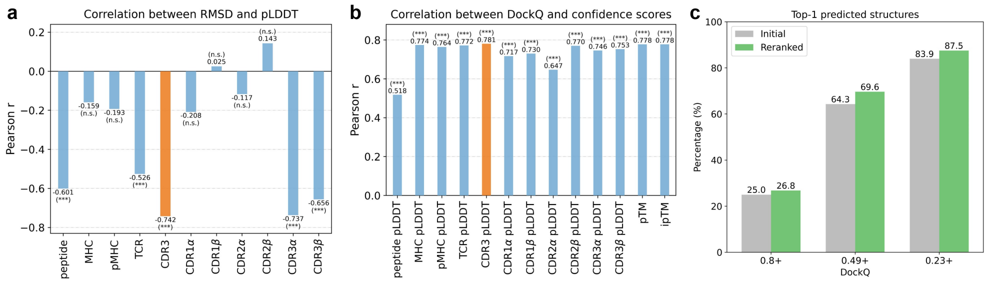

Supplementary Fig. 9 CDR3 pLDDT-based analysis of AlphaFold3 for class I TCR–pMHC. a. Correlation between pLDDT scores and RMSD of different subregions. CDR3 pLDDT and CDR3 RMSD exhibit the highest correlation compared to other components. Statistical significance is indicated as: * p<0.05; ** p<0.01; *** p<0.001; n.s., not significant. **b.** Correlation between confidence scores (including pLDDT scores of different subregions and pTM/ipTM) and DockQ. CDR3 pLDDT also shows the strongest correlation. **c.** Comparison of DockQ distributions for Top-1 predicted structures before and after reranking AlphaFold3's five predicted results using CDR3 pLDDT. Reranking improves the overall docking quality of Top-1 predictions.

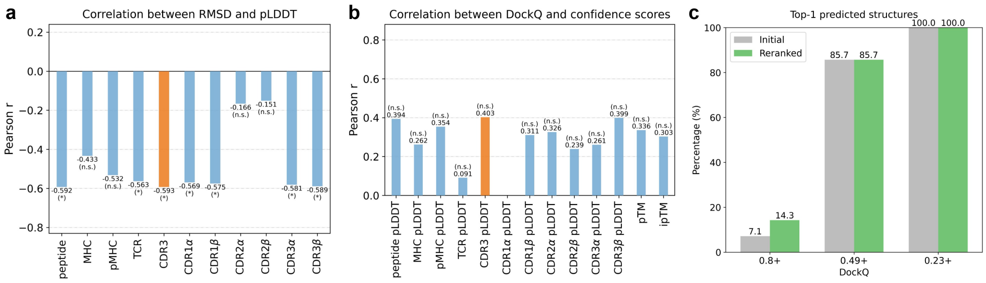

Supplementary Fig. 10 CDR3 pLDDT-based analysis of AlphaFold3 for class II TCR–pMHC. a. Correlation between pLDDT scores and RMSD of different subregions. CDR3 pLDDT and CDR3 RMSD exhibit the highest correlation compared to other components. Statistical significance is indicated as: * $p<0.05$; ** $p<0.01$; *** $p<0.001$; n.s., not significant. **b.** Correlation between confidence scores (including pLDDT scores of different subregions and pTM/ipTM) and DockQ. CDR3 pLDDT also shows the strongest correlation. **c.** Comparison of DockQ distributions for Top-1 predicted structures before and after reranking AlphaFold3's five predicted results using CDR3 pLDDT. Reranking improves the high docking quality (i.e., DockQ > 0.8) of Top-1 predictions.

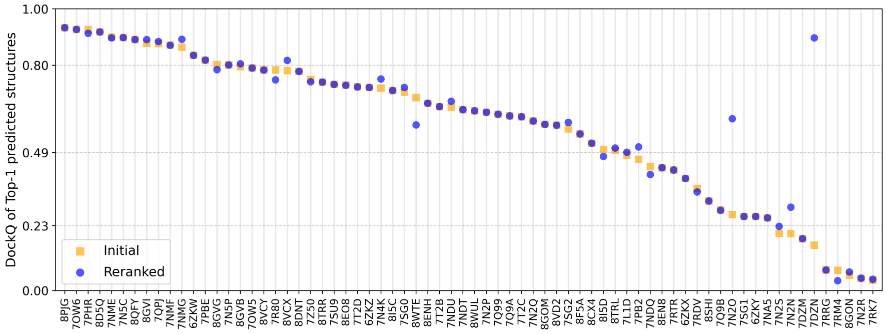

Supplementary Fig. 11 Changes in DockQ scores for Top-1 predicted structures across all AF3 samples after CDR3 pLDDT-based reranking. After reranking 70 samples, 40 DockQ scores remained unchanged; 11 showed slight decreases that did not affect docking quality levels; 19 improved, with two (7N2O and 7DZN) increasing by over 0.3.

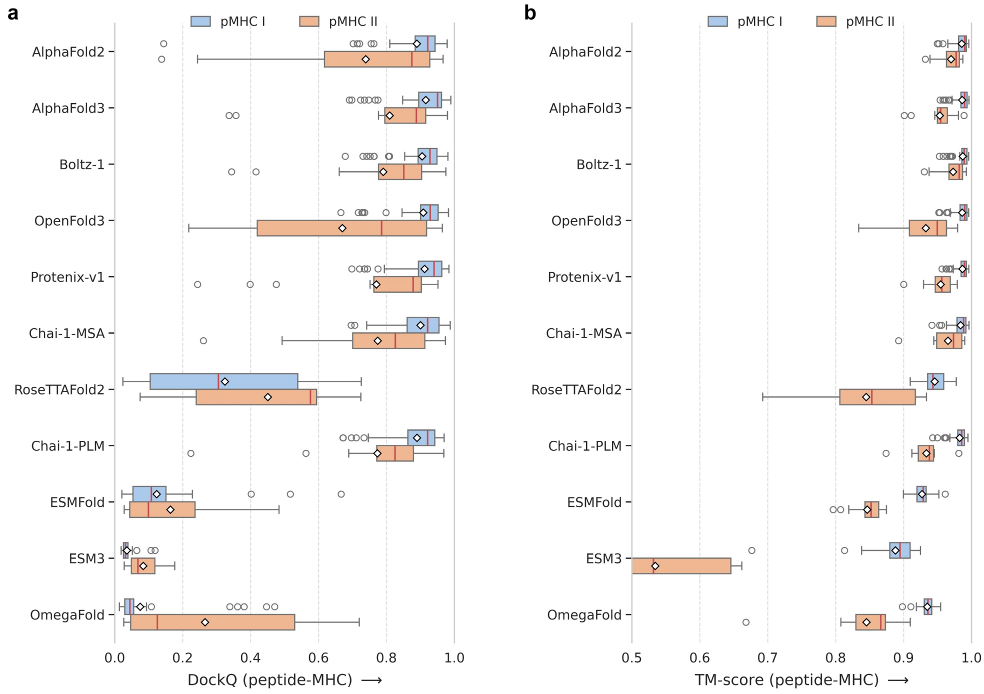

Supplementary Fig. 12 Structural modeling evaluation of pMHC (peptide–MHC) in TCR–pMHC. a. Peptide–MHC DockQ distribution. AlphaFold3 achieved the highest median DockQ scores for TCR–pMHC complexes in both class I (0.950) and class II (0.889), as well as the highest mean DockQ scores for class I (0.916) and class II (0.809). **b.** Peptide–MHC TM-score distribution. AlphaFold2 achieved the highest median TM-score (0.9902) for class I TCR–pMHC complexes, while Boltz-1 achieved the highest median TM-score (0.9819) for class II complexes. Boltz-1 also achieved the highest mean TM-scores for both class I (0.9871) and class II (0.9729) TCR–pMHC complexes.

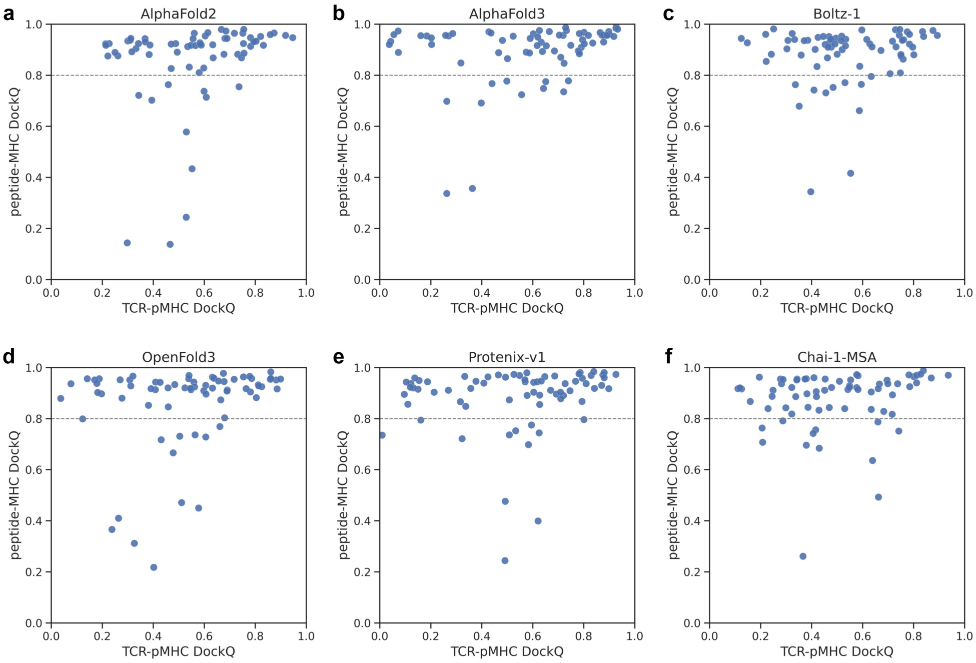

Supplementary Fig. 13 Comparison of pMHC DockQ and TCR–pMHC DockQ across different models. a. AlphaFold2. **b.** AlphaFold3. **c.** Boltz-1. **d.** OpenFold3. **e.** Protenix-v1. **f.** Chai-1-MSA.

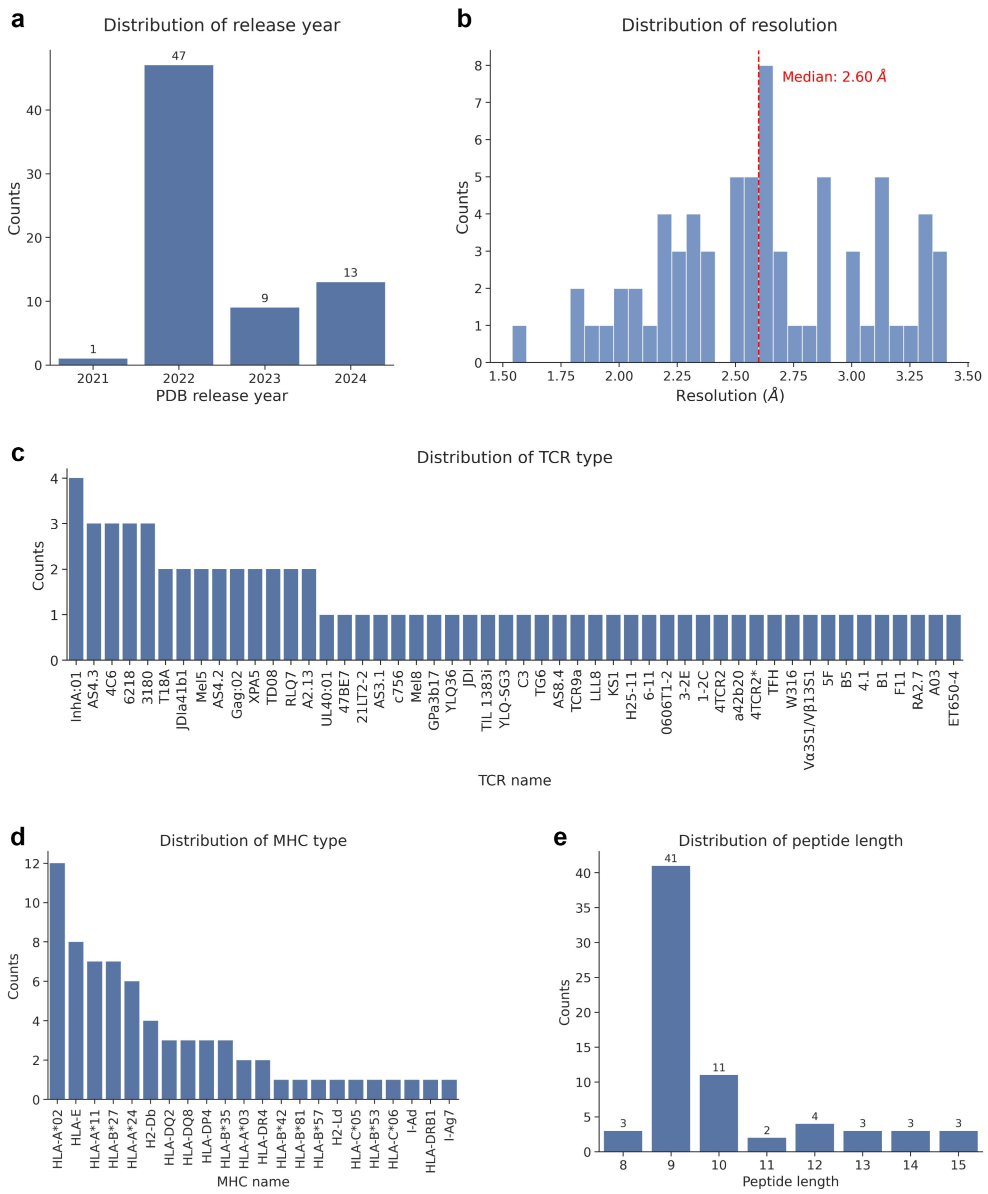

Supplementary Fig. 14 Detailed statistics of TCR–pMHC structures in benchmark data. a. Distribution of release year. **b.** Distribution of resolution. **c.** Distribution of TCR type. **d.** Distribution of MHC type. **e.** Distribution of peptide length.

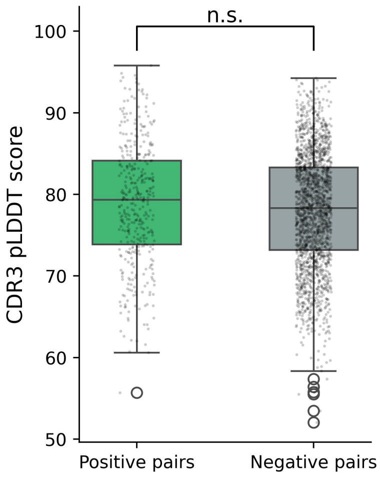

Supplementary Fig. 15 Differences in CDR3 pLDDT distribution between positive TCR–pMHC pairs and negative TCR–pMHC pairs in the STAPLER [5] test set after folding prediction. The STAPLER test set contains 396 positive and 2,011 negative TCR–pMHC sequence pairs. Structure prediction was performed using ColabFold-AF2. The median CDR3 pLDDT score was higher in positive samples than in negative samples, but the overall distribution showed no statistically significant difference (p-value=0.06). “n.s.” donates “not significant”.

3 Supplementary Tables

	AF2	AF3	Boltz-1	OpenFold3	Protenix-v1	Chai-1-MSA	RF2	Chai-1-PLM	ESMFold	ESM3	OmegaFold	AlphaRED	AF2+HDOCK
Docking Metrics													
DockQ ↑	*0.582*	**0.640**	0.519	0.554	0.581	0.461	0.129	0.546	0.038	0.021	0.028	0.472	0.022
Fnat ↑	0.600	**0.653**	0.563	0.586	*0.603*	0.500	0.077	0.542	0.000	0.000	0.000	0.391	0.000
iRMS ↓	2.093	**1.539**	2.361	2.110	*1.967*	2.314	7.386	2.045	14.002	20.625	12.503	2.530	16.675
LRMS ↓	4.531	**3.643**	5.046	5.014	*4.361*	6.720	13.224	5.176	28.151	36.936	29.910	5.111	41.811
RMSD ↓													
TCR–pMHC	*2.226*	**1.812**	2.389	2.456	2.392	2.588	7.016	2.503	19.443	19.721	16.595	2.481	18.639
peptide	0.719	**0.499**	0.748	0.634	*0.594*	0.686	2.342	0.721	2.383	3.457	2.428	0.732	0.719
MHC	0.551	*0.538*	**0.507**	0.567	0.549	0.587	0.923	0.701	1.129	1.467	0.797	0.774	0.551
pMHC	0.642	**0.589**	*0.595*	0.632	0.602	0.657	2.506	0.749	4.841	7.123	6.023	0.858	0.642
TCR$\alpha\beta$	1.054	0.973	**0.955**	0.968	*0.961*	1.064	1.321	1.117	1.309	14.246	1.365	1.173	1.054
CDR3$\alpha\beta$	1.492	*1.280*	1.428	1.353	**1.250**	1.729	1.804	1.501	1.761	14.227	1.834	1.549	1.492
CDR1α	0.442	0.395	0.373	**0.351**	*0.362*	0.376	0.496	0.422	0.595	0.753	0.541	0.563	0.442
CDR1β	0.280	0.253	0.256	**0.237**	0.263	*0.245*	0.323	0.290	0.321	0.476	0.287	0.430	0.280
CDR2α	0.352	0.311	0.312	**0.294**	*0.302*	0.315	0.348	0.364	0.406	0.587	0.432	0.563	0.352
CDR2β	0.278	**0.226**	0.236	0.240	*0.227*	0.240	0.336	0.272	0.320	0.545	0.308	0.409	0.278
CDR3α	0.950	*0.877*	1.084	**0.841**	0.940	1.167	1.093	1.079	1.123	1.231	1.183	0.969	0.950
CDR3β	0.839	*0.743*	0.771	0.753	**0.683**	1.032	1.172	0.815	1.018	1.209	1.368	0.856	0.839
TM-score ↑													
TCR–pMHC	0.923	**0.956**	0.916	0.914	*0.933*	0.902	0.642	0.916	0.546	0.438	0.558	0.908	0.550
peptide	0.525	**0.661**	0.552	0.553	*0.593*	0.543	0.254	0.542	0.248	0.131	0.289	0.520	0.525
MHC	*0.989*	*0.989*	**0.990**	0.988	*0.989*	*0.989*	0.971	0.985	0.961	0.929	0.976	0.978	*0.989*
pMHC	0.987	**0.989**	**0.989**	0.987	*0.988*	0.986	0.940	0.982	0.927	0.887	0.933	0.977	0.987
TCR$\alpha\beta$	0.969	*0.973*	**0.975**	*0.973*	**0.975**	0.970	0.956	0.969	0.958	0.515	0.951	0.962	0.969
CDR3$\alpha\beta$	0.622	0.641	*0.652*	0.634	**0.672**	0.595	0.581	0.609	0.590	0.327	0.548	0.612	0.622
CDR1α	0.647	0.691	0.710	*0.728*	**0.735**	0.701	0.593	0.673	0.554	0.469	0.562	0.558	0.647
CDR1β	0.786	0.825	0.818	**0.838**	0.809	*0.828*	0.743	0.779	0.751	0.604	0.778	0.663	0.786
CDR2α	0.734	*0.767*	0.758	**0.776**	0.765	0.765	0.727	0.729	0.683	0.520	0.675	0.603	0.734
CDR2β	0.814	**0.855**	0.836	0.834	*0.844*	0.831	0.746	0.804	0.753	0.566	0.763	0.657	0.814
CDR3α	0.480	*0.509*	0.473	**0.524**	0.500	0.475	0.441	0.484	0.457	0.370	0.437	0.449	0.480
CDR3β	0.570	0.564	*0.572*	*0.572*	**0.579**	0.559	0.484	0.553	0.521	0.398	0.501	0.500	0.570

Supplementary Table 1 Median results of 13 representative models on 70 benchmark cases. Bold and *underline* indicate the best and second metrics for evaluated models, respectively. 'AF2', 'AF3', and 'RF2' denote AlphaFold2, AlphaFold3, and RoseTTAFold2, respectively.

	AF2	AF3	Boltz-1	OpenFold3	Protenix-v1	Chai-1-MSA	RF2	Chai-1-PLM	ESMFold	ESM3	OmegaFold	AlphaRED	AF2+HDOCK
Docking Metrics													
DockQ ↑	*0.563*	**0.585**	0.543	0.530	0.536	0.494	0.191	0.514	0.077	0.022	0.061	0.453	0.233
Fnat ↑	*0.563*	**0.567**	0.537	0.536	0.531	0.485	0.138	0.517	0.043	0.011	0.035	0.403	0.239
iRMS ↓	*2.332*	2.717	**2.330**	2.845	2.978	2.660	7.477	2.547	14.734	20.536	14.063	4.324	13.204
LRMS ↓	**5.168**	6.015	*5.421*	6.394	6.631	6.531	15.625	6.283	36.745	39.337	38.027	9.936	31.949
RMSD ↓													
TCR–pMHC	*2.855*	3.554	**2.824**	3.361	5.010	3.252	9.727	3.046	16.747	19.772	15.214	4.528	13.978
peptide	1.048	**0.862**	1.004	0.973	*0.925*	1.002	2.640	1.030	2.671	3.734	2.681	1.052	1.048
MHC	*0.616*	0.905	**0.596**	0.920	0.822	0.767	1.202	1.077	1.544	2.978	1.327	0.868	*0.616*
pMHC	*0.804*	0.956	**0.712**	1.103	0.910	0.868	3.005	1.181	4.852	7.604	6.017	1.007	*0.804*
TCR$\alpha\beta$	1.181	**1.057**	1.076	1.104	*1.063*	1.176	1.469	1.205	1.667	14.272	1.728	1.283	1.181
CDR3$\alpha\beta$	1.543	*1.485*	1.639	1.539	**1.463**	1.778	1.904	1.665	2.175	14.144	2.122	1.628	1.543
CDR1α	0.512	0.473	0.452	**0.441**	*0.444*	0.489	0.628	0.575	0.732	0.838	0.714	0.609	0.512
CDR1β	0.297	*0.282*	0.284	**0.274**	0.285	0.289	0.355	0.309	0.343	0.581	0.336	0.428	0.297
CDR2α	0.543	*0.430*	0.435	**0.395**	0.447	0.483	0.486	0.527	0.834	0.717	0.673	0.653	0.543
CDR2β	0.323	*0.278*	0.281	0.281	**0.276**	0.330	0.429	0.355	0.370	0.616	0.413	0.450	0.323
CDR3α	1.149	**1.109**	1.227	1.171	*1.115*	1.305	1.356	1.243	1.292	1.391	1.346	1.203	1.149
CDR3β	1.065	1.030	1.136	*1.026*	**1.007**	1.233	1.337	1.152	1.344	1.445	1.398	1.116	1.065
TM-score ↑													
TCR–pMHC	*0.901*	0.895	**0.904**	0.879	0.866	0.878	0.705	0.886	0.597	0.417	0.598	0.859	0.674
peptide	0.550	**0.631**	0.556	0.548	*0.588*	0.556	0.270	0.539	0.259	0.153	0.285	0.541	0.550
MHC	*0.986*	0.982	**0.988**	0.980	0.983	0.983	0.950	0.975	0.949	0.854	0.958	0.973	*0.986*
pMHC	*0.983*	0.980	**0.984**	0.976	0.980	0.980	0.926	0.973	0.911	0.817	0.917	0.970	*0.983*
TCR$\alpha\beta$	0.961	**0.970**	**0.970**	*0.967*	**0.970**	0.966	0.945	0.963	0.936	0.516	0.934	0.955	0.961
CDR3$\alpha\beta$	0.639	*0.656*	0.646	0.645	**0.664**	0.616	0.553	0.617	0.579	0.325	0.564	0.608	0.639
CDR1α	0.648	0.696	0.705	**0.714**	*0.710*	0.687	0.589	0.645	0.560	0.478	0.576	0.582	0.648
CDR1β	0.780	*0.800*	0.798	**0.804**	0.793	0.793	0.727	0.770	0.734	0.583	0.755	0.674	0.780
CDR2α	0.703	0.741	0.745	**0.760**	*0.749*	0.730	0.698	0.697	0.633	0.525	0.655	0.606	0.703
CDR2β	0.783	**0.819**	0.812	*0.816*	**0.819**	0.800	0.713	0.777	0.736	0.557	0.737	0.666	0.783
CDR3α	0.508	**0.534**	0.501	0.524	*0.526*	0.485	0.441	0.489	0.453	0.381	0.449	0.472	0.508
CDR3β	0.582	*0.599*	0.589	0.591	**0.607**	0.584	0.493	0.573	0.525	0.399	0.522	0.525	0.582

Supplementary Table 2 Mean results of 13 representative models on 70 benchmark cases. Bold and *underline* indicate the best and second metrics for evaluated models, respectively. 'AF2', 'AF3', and 'RF2' denote AlphaFold2, AlphaFold3, and RoseTTAFold2, respectively.

Model	Total inference time per sample (seconds)
AlphaFold2	7613.6
ALphaFold3	110.9
Boltz-1	115.3
OpenFold3	118
Protenix-v1	231.4
Chai-1-MSA	310.8
RoseTTAFold2	647
Chai-1-PLM	140.2
ESMFold	30.1
ESM3	0.3
OmegaFold	171.2
AlphaRED	23636.9
AF2+HDOCK	8247.8

Supplementary Table 3 Average total inference time across different models. Note that AlphaFold3 and Chai-1 were accessed via web servers, while other models ran locally.

	AF2 (No_MSA)	AF2 (Full_DB)	AF2 (Reduced_DB)	TCRmodel2	ColabFold
Docking Metrics					
DockQ ↑	0.016	0.582	0.539	**0.595**	*0.583*
Fnat ↑	0.000	**0.600**	0.552	*0.575*	0.571
iRMS ↓	19.791	2.093	2.331	**1.996**	*1.997*
LRMS ↓	44.889	4.531	5.033	**4.453**	*4.472*
RMSD ↓					
TCR–pMHC	20.711	*2.226*	2.289	**2.043**	2.300
peptide	3.587	0.719	0.722	**0.492**	*0.585*
MHC	0.598	0.551	*0.522*	**0.497**	0.533
pMHC	5.916	0.642	0.671	**0.549**	*0.626*
TCR$\alpha\beta$	5.397	1.054	1.054	*0.996*	**0.891**
CDR3$\alpha\beta$	5.340	1.492	1.445	**1.230**	*1.250*
CDR1α	0.366	0.442	0.440	*0.335*	**0.333**
CDR1β	0.269	0.280	0.281	**0.256**	*0.259*
CDR2α	0.308	0.352	0.359	*0.301*	**0.298**
CDR2β	0.238	0.278	0.260	*0.226*	**0.223**
CDR3α	1.270	0.950	1.058	*0.889*	**0.818**
CDR3β	0.991	0.839	0.829	*0.782*	**0.770**
TM-score ↑					
TCR–pMHC	0.489	0.923	0.915	**0.935**	*0.926*
peptide	0.243	0.525	0.522	**0.625**	*0.606*
MHC	0.987	0.989	*0.990*	**0.991**	*0.990*
pMHC	0.944	0.987	0.986	**0.990**	*0.988*
TCR$\alpha\beta$	0.610	0.969	0.970	*0.973*	**0.977**
CDR3$\alpha\beta$	0.410	0.622	0.614	***0.655***	***0.690***
CDR1α	0.709	0.647	0.658	**0.752**	*0.735*
CDR1β	0.801	0.786	0.794	**0.818**	*0.812*
CDR2α	0.773	0.734	0.719	*0.793*	**0.801**
CDR2β	0.839	0.814	0.825	*0.849*	**0.853**
CDR3α	0.439	0.480	0.481	*0.485*	**0.505**
CDR3β	0.542	0.570	0.557	*0.573*	**0.582**

Supplementary Table 4 Median results of AlphaFold2 with different MSA generation strategies on 70 benchmark cases. **Bold** and *underline* indicate the best and second metrics for evaluated models, respectively.

PDB	Mutation	$\Delta\Delta G$	mutant			wild-type		
			CDR3 pLDDT	ranking_score	ipTM	CDR3 pLDDT	ranking_score	ipTM
1AO7	DD99A	0.78	97.54	0.94	0.93	98.13	0.95	0.94
1AO7	DD99N	2.46	98.07	0.95	0.94	98.13	0.95	0.94
1AO7	SD100A	-0.24	97.86	0.95	0.93	98.13	0.95	0.94
1AO7	SD100N	1.91	97.63	0.95	0.93	98.13	0.95	0.94
1AO7	SD100T	-0.49	98.2	0.95	0.93	98.13	0.95	0.94
1AO7	SD100Y	2.32	98.5	0.95	0.94	98.13	0.95	0.94
1AO7	WD101A	2.22	97.97	0.95	0.94	98.13	0.95	0.94
1AO7	AE99K	0.06	97.83	0.96	0.94	98.13	0.95	0.94
1AO7	AE99M	-0.31	98.04	0.95	0.94	98.13	0.95	0.94
1AO7	GE100S	-0.33	98.15	0.95	0.94	98.13	0.95	0.94
1AO7	GE101A	-1.09	98.57	0.95	0.94	98.13	0.95	0.94
1AO7	GE101P	0.45	96.73	0.94	0.92	98.13	0.95	0.94
1AO7	RE102A	0.06	97.95	0.95	0.93	98.13	0.95	0.94
1AO7	RE102P	-1.94	97.87	0.95	0.94	98.13	0.95	0.94
1AO7	RE102Q	0.41	97.54	0.94	0.93	98.13	0.95	0.94
3QDG	FE100W	0.93	96.83	0.94	0.92	97.13	0.94	0.92
3QDG	FE100Y	1.39	95.7	0.94	0.92	97.13	0.94	0.92
3QDG	TE102F	-0.04	97.16	0.94	0.93	97.13	0.94	0.92
3QDJ	FE100W	0.39	96.09	0.93	0.92	96.64	0.94	0.93
3QDJ	FE100Y	0.78	95.77	0.94	0.92	96.64	0.94	0.93
3QDJ	TE102F	-0.28	97.06	0.94	0.92	96.64	0.94	0.93

Supplementary Table 5 $\Delta\Delta G$-SKEMPI dataset details and confidence scores for wild-type and mutant structures predicted by AlphaFold3. Note that for each mutation, the first letter denotes the wild-type amino acid, the second letter denotes the chain ID in the PDB entry, the number indicates the position number specified by the authors in the PDB entry, and the final letter denotes the mutated amino acid.

PDB	Mutation	$\Delta\Delta G$ (FoldX)	mutant			wild-type		
			CDR3 pLDDT	ranking_score	ipTM	CDR3 pLDDT	ranking_score	ipTM
6ZKX	GE112A	3.12	94.02	0.84	0.86	91.26	0.77	0.79
6ZKX	GE112E	9.71	91.70	0.79	0.81	91.26	0.77	0.79
6ZKX	GE112K	8.10	83.43	0.46	0.50	91.26	0.77	0.79
6ZKX	GE112R	8.90	82.29	0.48	0.52	91.26	0.77	0.79
6ZKX	GE112S	2.95	90.59	0.77	0.80	91.26	0.77	0.79
6ZKX	RE111A	6.19	81.35	0.68	0.71	91.26	0.77	0.79
6ZKX	RE111E	7.46	95.73	0.88	0.90	91.26	0.77	0.79
6ZKX	RE111K	5.15	82.15	0.46	0.50	91.26	0.77	0.79
6ZKX	RE111Q	5.97	85.40	0.63	0.66	91.26	0.77	0.79
6ZKX	RE111T	7.09	81.17	0.48	0.52	91.26	0.77	0.79
6ZKX	RE113A	2.28	92.53	0.79	0.82	91.26	0.77	0.79
6ZKX	RE113E	2.35	76.70	0.49	0.53	91.26	0.77	0.79
6ZKX	RE113H	2.46	85.07	0.53	0.57	91.26	0.77	0.79
6ZKX	RE113Q	2.12	85.14	0.51	0.55	91.26	0.77	0.79
6ZKX	RE113W	1.21	83.65	0.64	0.67	91.26	0.77	0.79
6ZKX	SE112D	-0.25	92.17	0.76	0.79	91.26	0.77	0.79
6ZKX	SE112E	0.06	88.23	0.80	0.82	91.26	0.77	0.79
6ZKX	SE112K	-0.35	93.59	0.83	0.85	91.26	0.77	0.79
6ZKX	SE112R	-0.85	88.44	0.58	0.62	91.26	0.77	0.79
6ZKX	SE112Y	-0.21	93.05	0.84	0.86	91.26	0.77	0.79
7DZM	ID99C	1.96	96.73	0.92	0.93	77.66	0.62	0.64
7DZM	ID99G	1.96	95.72	0.91	0.92	77.66	0.62	0.64
7DZM	ID99L	0.21	78.47	0.64	0.66	77.66	0.62	0.64
7DZM	ID99P	0.90	74.70	0.59	0.61	77.66	0.62	0.64
7DZM	ID99T	2.57	96.55	0.92	0.93	77.66	0.62	0.64
7DZN	ID99E	1.36	73.18	0.58	0.60	76.53	0.58	0.60
7DZN	ID99F	3.67	95.60	0.90	0.91	76.53	0.58	0.60
7DZN	ID99L	-0.35	80.87	0.58	0.61	76.53	0.58	0.60
7DZN	ID99N	1.61	94.38	0.87	0.89	76.53	0.58	0.60
7DZN	ID99R	1.50	74.92	0.57	0.60	76.53	0.58	0.60
7N2O	FE98C	3.13	80.59	0.70	0.72	81.33	0.72	0.74
7N2O	FE98D	4.90	72.87	0.48	0.51	81.33	0.72	0.74
7N2O	FE98E	2.44	77.23	0.51	0.53	81.33	0.72	0.74
7N2O	FE98N	2.68	87.50	0.72	0.74	81.33	0.72	0.74
7N2O	FE98Q	2.23	79.55	0.54	0.57	81.33	0.72	0.74
7N2O	SE99C	0.05	91.85	0.80	0.82	81.33	0.72	0.74
7N2O	SE99D	6.16	75.87	0.50	0.53	81.33	0.72	0.74
7N2O	SE99G	1.19	81.08	0.73	0.75	81.33	0.72	0.74
7N2O	SE99K	3.33	76.89	0.48	0.51	81.33	0.72	0.74
7N2O	SE99N	3.25	76.71	0.66	0.68	81.33	0.72	0.74
7N2O	TE100C	-0.76	81.02	0.70	0.71	81.33	0.72	0.74
7N2O	TE100E	0.45	78.55	0.61	0.63	81.33	0.72	0.74
7N2O	TE100K	-0.71	85.20	0.71	0.73	81.33	0.72	0.74
7N2O	TE100P	-0.97	77.18	0.66	0.68	81.33	0.72	0.74
7N2O	TE100Q	-0.94	79.05	0.67	0.68	81.33	0.72	0.74
7NA5	GE100A	1.96	89.42	0.62	0.65	93.77	0.79	0.81
7NA5	GE100C	6.58	90.53	0.67	0.69	93.77	0.79	0.81
7NA5	GE100P	3.30	90.56	0.69	0.71	93.77	0.79	0.81
7NA5	GE100Q	9.25	83.15	0.62	0.64	93.77	0.79	0.81
7NA5	GE100S	6.89	88.48	0.65	0.68	93.77	0.79	0.81
7NA5	GE99L	7.69	85.36	0.65	0.67	93.77	0.79	0.81
7NA5	GE99M	9.48	85.36	0.59	0.62	93.77	0.79	0.81
7NA5	GE99S	8.04	86.63	0.59	0.61	93.77	0.79	0.81
7NA5	GE99T	8.99	85.94	0.56	0.59	93.77	0.79	0.81
7NA5	GE99V	8.46	89.44	0.57	0.60	93.77	0.79	0.81
7NA5	YE101I	3.58	88.63	0.61	0.64	93.77	0.79	0.81
7NA5	YE101K	4.12	89.23	0.58	0.61	93.77	0.79	0.81
7NA5	YE101P	4.78	85.95	0.64	0.66	93.77	0.79	0.81
7NA5	YE101V	4.65	88.96	0.62	0.65	93.77	0.79	0.81
7NA5	YE101W	2.64	89.79	0.68	0.70	93.77	0.79	0.81

Supplementary Table 6 **$\Delta\Delta G$-FoldX dataset details and confidence scores for wild-type and mutant structures predicted by AlphaFold3.**

	AUROC	AUPRC
pTM	0.526	0.231
ipTM	0.531	0.229
Ranking score	0.527	0.229
CDR3 pLDDT	0.545	0.254
STAPLER [5]	0.642	0.363

Supplementary Table 7 Classification performance of the STAPLER test set. The STAPLER test set contains 396 positive and 2,011 negative TCR–pMHC sequence pairs. Structural predictions were performed using ColabFold-AF2, with pTM, ipTM, ranking score, and CDR3 pLDDT extracted as scores for each sample. AUROC [6] and AUPRC [7] were calculated as evaluation metrics. CDR3 pLDDT scoring outperformed other confidence scores, but showed a gap compared to the specialized STAPLER [5] model.

References

[1] Yin, R. *et al.* Tcrmodel2: high-resolution modeling of t cell receptor recognition using deep learning. *Nucleic Acids Research* **51**, W569–W576 (2023).

[2] Mirdita, M. *et al.* Colabfold: making protein folding accessible to all. *Nature methods* **19**, 679–682 (2022).

[3] Steinegger, M. & Söding, J. Mmseqs2 enables sensitive protein sequence searching for the analysis of massive data sets. *Nature biotechnology* **35**, 1026–1028 (2017).

[4] Mirdita, M., Steinegger, M. & Söding, J. Mmseqs2 desktop and local web server app for fast, interactive sequence searches. *Bioinformatics* **35**, 2856–2858 (2019).

[5] Kwee, B. P. *et al.* Stapler: efficient learning of tcr-peptide specificity prediction from full-length tcr-peptide data. *BioRxiv* 2023–04 (2023).

[6] Fawcett, T. An introduction to roc analysis. *Pattern recognition letters* **27**, 861–874 (2006).

[7] Davis, J. & Goadrich, M. *The relationship between precision-recall and roc curves*, 233–240 (2006).
